# Antimicrobial Properties of Polymer-Based Nanocomposites Modified by Nanoparticles Produced by Green Chemistry

**DOI:** 10.3390/ma19020251

**Published:** 2026-01-08

**Authors:** Anna Wasilewska, Magda Bielicka, Urszula Klekotka, Grzegorz Markiewicz, Marek Jałbrzykowski, Wioleta Lewandowska, Izabela Swiecicka, Beata Kalska-Szostko

**Affiliations:** 1 Doctoral School, University of Bialystok, Ciołkowskiego 1K 1, 15-245 Białystok, Poland; 2 Faculty of Chemistry, University of Białystok, Ciołkowskiego 1K 1, 15-245 Białystok, Poland; 3 Faculty of Mechanical Engineering, Bialystok University of Technology, Wiejska 45C, 15-351 Białystok, Poland; 4 Faculty of Biology, University of Bialystok, Ciolkowskiego 1J, 15-245 Bialystok, Poland; 5 Laboratory of Applied Microbiology, University of Bialystok, Ciolkowskiego 1J, 15-245 Bialystok, Poland

**Keywords:** Ag NPs, Cu NPs, green chemistry, composites, antimicrobial activity

## Abstract

A significant driving force in nanotechnology development is the environmentally friendly synthesis of nanomaterials using natural extracts as reducing and stabilizing agents. In this study, silver and copper nanoparticles were synthesized and compared using two approaches: (1) a green synthesis pathway employing beetroot extract as a natural bio-reductant and stabilizer, and (2) a conventional chemical reduction method. The resulting nanoparticles were extensively characterized using transmission electron microscopy (TEM), X-ray diffraction (XRD), UV-Vis spectroscopy, and dynamic light scattering (DLS). The study revealed that the green synthesis route produced nanoparticles with well-defined morphology, high stability, and strong antimicrobial potential, outperforming those obtained via conventional chemical synthesis. Copper nanoparticles synthesized using beetroot extract exhibited particularly enhanced fungicidal and bactericidal properties, demonstrating the effectiveness of plant-based reducing agents in producing functional nanostructures. To further evaluate potential applications, the green-synthesized nanoparticles were incorporated into a polypropylene matrix, confirming their integrity and activity within the composite system. This work emphasizes the role of green synthesis in designing high-performance nanomaterials and highlights the promising capabilities of beetroot extract as a sustainable and efficient reducing and stabilizing medium for silver and copper nanoparticle production.

## 1. Introduction

In recent years, the subject of polymer-based composites reinforced with nanoparticles has attracted the interest of many scientists [1,2,3]. Polymer nanocomposites are an innovative class of materials that exhibit improved functional properties due to the addition of very small particles (10^−7^ to 10^−9^ m) to the polymer matrix [2]. These include strength, mechanical resistance, flexibility, abrasion, mega-volt insulation, electrical conductivity, barrier to gas permeability, fire resistance, and scratch resistance, etc. [3]. Introducing a nanofiller changes the properties of the polymer, giving the nanocomposite attractive features that can be used, for example, in packaging design, medical applications, and many others [4]. Nanoparticles introduced into the polymer can have various shapes, e.g., nanofibers, nanotubes, nanorods, nanoplates, or nanograins [5]. Typically, the surfaces of the nanoparticles are modified to improve the adhesion of the polymer to the nanoparticles [6], which is a critical step to obtain new or maintain attractive properties of the nanocomposite [7]. An exciting direction in developing new nanocomposites is related to one possessing antimicrobial properties [8].

One of the types of polymer-inorganic nanocomposites is a system made of a polymer matrix and inorganic nanoparticles embedded in its structure [9]. Polymers used to create hybrid systems are divided in terms of their origin into two main groups: synthetic polymers and natural polymers (biopolymers, e.g., polysaccharides, lipids) [10]. Synthetic polymers can be precisely designed, and their properties are well adapted to the needs. On the other hand, biopolymers have a strictly defined structure, but their great diversity, internal biocompatibility, and biodegradability compensate for the lower possibility of manipulating the properties. Polyvinylpyrrolidone (PVP)-coated nanoparticles are characterized by good solubility in water and some organic solvents, thanks to which phase transfer between these solvents is possible [11]. For example, PVP-coated silver nanoparticles show high stability and biocompatibility [12]. Attention should also be paid to nanoparticles introduced into the polymer matrix. The presented research is focused on nanoparticles fabricated both by traditional and ‘green’ synthesis methods and checking whether synthesis routine affects the morphological properties and stability of composites, and their antimicrobial potential [13], since an exciting direction concerns usage of nanocomposites as antimicrobial agents [8].

Green synthesis of nanoparticles, based on the principles of green chemistry, aims to reduce hazardous substances, minimize energy consumption, and employ renewable or naturally derived reagents. Plant extracts are increasingly used as reducing and stabilizing agents, offering a non-toxic and low-cost alternative to chemical reduction. However, despite the growing number of studies, many plant sources remain underexplored. Beetroot extract is one such promising but insufficiently studied bioreductant. It contains betalains and phenolic compounds capable of reducing and stabilizing metal nanoparticles. Unlike commonly used extracts (e.g., green tea or neem), beetroot is inexpensive, widely available, and offers potential for developing new eco-friendly synthesis pathways.

Metal/polymer nanocomposites, due to the high dispersion potential of the metal clusters, the large nanoparticle/polymer contact surface ensures high reactivity and, ultimately, good metal-releasing properties. That can be attractive, for example, in plant cultivation and textile production, etc. [14,15]. Although much work has been devoted to synthesizing, researching, and developing metal-based nanocomposite materials, little is known about their antifungal and/or antibacterial properties [16]. This was the motivation to undertake the presented research. Prospective use of the developed nanomaterials embedded in the polymer can be envisaged in the preparation of antifungal coatings for use in, for example, the textile industry, gardening, etc. [17,18]. The dispersion of nanoparticles in the polymer improves the properties of the final nanocomposite. A significant challenge in preparing polymer nanocomposites is obtaining a homogeneous distribution of nanoparticles in the polymer matrix, knowing that better dispersion of nanoparticles allows for more significant matrix-nanoparticle interaction, which can be responsible for improving the biostatic properties of the material. Copper nanoparticles have a solid inhibitory and antifungal effect on a broad spectrum of fungi [19,20]. Nanosilver is known for its antibacterial, antifungal, and antiviral effects [21]. Currently, colloidal silver is used externally, i.e., on the skin and mucous membranes. This compound is often used in ophthalmology to treat bacterial conjunctivitis [22]. It is also possible to modify materials by depositing silver nanoparticles on carriers or covering various surfaces with them so that they have biocidal, deodorizing, antistatic, and impregnating properties [22,23,24]. Research on polymer nanocomposites has developed rapidly over the last ten years [25]. Such nanocomposites have attracted the attention of industrial researchers due to their remarkable electrical, thermal, chemical, and biological properties and potential applications in various industrial sectors [24,25]. Despite rising interest in green synthesis, the current literature lacks systematic comparisons between nanoparticles produced by different green reducing agents and their chemically synthesized analogues. In particular, there is limited information on how beetroot extract influences nanoparticle formation, stability, and subsequent performance in polymer matrices.

Copper and silver nanoparticles mixed with a polymer can lead to more efficient antimicrobial properties [26]. Surface modification generally reduces the surface energy of nanoparticles, improving their affinity to the polymer matrix, which affects the processing of such a composite [27,28,29]. The present study compares the developed method of adding the nanoparticles produced by the ‘green chemistry’ routine and those synthesized by the traditional method (chemical reduction using borohydride) to the polymer. This work aimed to explore underused or less commonly studied natural reducing agents. Beetroot is not as widely used as, for example, green tea or neem, so it offers a novel contribution and comparative potential. The resulting product is assumed to be characterized by antifungal or antibacterial activity. The product’s safety and durability are critical parameters that allow application in the integrated cultivation of vegetables or fruits, e.g., when creating agro textiles [30], or can be used as an additive to any textile products that require antibacterial or antifungal properties. Therefore, the research results can increase the environmental sustainability of nanoparticle production processes, which, in turn, suggests many possible applications, including medicine or engineering [31].

Synthesized by two methods, the nanoparticles were embedded into a polymer and then used for the measurements. The described method consists of adding to the machine at the same time polymer (polypropylene (PP)) and a liquid with nanoparticles produced by ‘green chemistry’ and those fabricated by chemical reduction. Overall, using PP allows us to test the integration of green-synthesized nanoparticles in a realistic and industrially relevant polymer matrix, highlighting the potential for commercial application of the developed nanomaterials. Despite growing interest in green synthesis of nanoparticles for incorporation into polymer composites, significant research gaps remain. In particular, existing studies often lack detailed comparisons of the advantages of specific plant extracts, such as beetroot, as reducing and stabilizing agents. This results in an incomplete understanding of how these bio-sources influence nanoparticle properties and their subsequent effects on the composite performance. Therefore, this study aims to address these gaps by systematically evaluating the use of beetroot extract for the green synthesis of silver and copper nanoparticles, and assessing their incorporation into polymer matrices. By thoroughly comparing chemically synthesized counterparts and analyzing the mechanistic aspects of nanoparticle formation and antimicrobial activity, the present work strengthens the motivation for applying green-synthesized nanoparticles in polymer composites.

## 2. Experimental

### 2.1. Materials

Silver nitrate (AgNO_3_, ≥99% purity) was purchased from POCH S.A. (Gliwice, Poland). Copper(II) nitrate (Cu(NO_3_)_2_·3H_2_O, ≥99% purity) was obtained from Chempur (Piekary Śląskie, Poland). Sodium dodecyl sulfate (SDS, ≥98% purity) and sodium borohydride (NaBH_4_, ≥98% purity) were supplied by Sigma-Aldrich (St. Louis, MO, USA) and used without further purification. Polypropylene (PP, isotactic grade) was provided by Thermo Scientific (Waltham, MA, USA). Fresh beetroot (*Beta vulgaris* L.) was sourced from a certified local supplier and processed immediately to prepare the natural reducing and stabilizing extract. All aqueous solutions were prepared using deionized water (18.2 MΩ·cm) produced by a Merck Millipore Direct-Q 3UV-R system (Merck KGaA, Darmstadt, Germany).

### 2.2. Materials and Characterization

Particle structure, morphology, diameter, and size distribution were identified by transmission electron microscopy (FEI Tecnai G2 X-TWIN at 200 kV, Hillsboro, OR, USA). Samples were prepared by dropping freshly prepared nanoparticle solution on the carbon-covered 400 mesh Cu grid and left for drying. Polymer homogeneity and particle incorporation into PP were verified by scanning electron microscopy (SEM—FEI INSPECT S60, Hillsboro, OR, USA) equipped with energy dispersive X-ray spectroscopy (EDX). The crystal structure and the resulting crystallites’ size were measured by X-ray diffraction using an XRD diffractometer Agilent Technologies SuperNova (Santa Clara, CA, USA) with a Mo microfocused source, K_α2_ = 0.71367. A small number of nanoparticles was placed on a nylon loop covered with highly viscous oil for each measurement. Dynamic Light Scattering estimated the hydrodynamic size using a DLS spectrometer, Nanoplus (Gerbrunn, Germany), which performed experiments in water at 20 ± 2 °C. A UV-Vis spectral analysis was performed to confirm the presence of nanoparticles in the obtained suspensions. The Able&Jasco V-670 spectrometer (JASCO Corporation, Tokyo, Japan) with a spectral range of 200–800 nm was used to detect the spectrum of particles dispersed in water.

### 2.3. Preparation of Beetroot Extract

**Figure 1:**
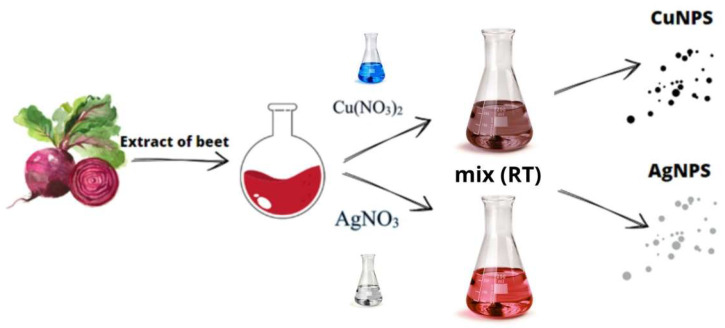
Schematic presentations of “green chemistry” synthesis in the presence of beetroot extract.

Beets were purchased from a local store to prepare the beetroot extract. First, the beetroot was washed twice with distilled water to remove any residue (soil, pesticides, etc.). The beetroot was then peeled, cut into several pieces, and added to the water, which was heated to 80 °C for 1 h. After cooling, the resulting mixture was filtered to obtain a clear liquid. The pH of the obtained juice was tested to be 4.2. Figure 1 shows a scheme for obtaining nanoparticles using the described “green chemistry” method.

### 2.4. Synthesis

Two synthesis methods were used to obtain silver and copper nanoparticles. The first was based on the chemical reduction of Ag and Cu particles using sodium borohydride and sodium lauryl sulfate (SLS). The second is a green synthesis that uses beetroot extract as a reducing and stabilizing agent for the obtained nanoparticles. The salt used as a precursor of silver ions was silver nitrate (AgNO_3_), and for the formation of copper nanoparticles, it was copper (II) nitrate (Cu(NO_3_)_2_). In both cases, the salt concentration used for synthesis was 0.1 mol/L.

Two nanoparticle formation processes for both silver and copper were followed:

(1) Sodium borohydride was used as a reducing agent, and sodium dodecyl sulfate was applied as a surface stabilizer. 25 mL of a salt solution and 0.015 g of sodium borohydride were mixed with 0.03 g of SDS-sodium dodecyl sulfate. The synthesis was carried out at room temperature. The solutions were stirred for 1 h. Acetone was used as a filtering solvent to obtain a clear solution. The product was centrifuged at 13,000 rpm for 30 min and then dried to a powder form. The synthesis was carried out at room temperature.

(2) Beetroot extract was used as a stabilizer and reducer. 100 g of beetroot was placed in 250 mL of distilled water. The whole process was carried out at 80 °C for 1 h. After heating, the entire thing was filtered and centrifuged twice to remove the beetroot residue. 25 mL of salt solution and 5 mL of beetroot extract were mixed for 1 h. The final synthesis step was carried out at room temperature. Acetone was used as a filtering solvent to obtain a clear solution. The produced liquid was centrifuged at 13,000 rpm for 30 min, and then the sediment was dried to a powder form.

The summary of the synthesis is presented in Table 1.

### 2.5. Composite Preparation

The following procedure was used to obtain polymer composites. An appropriate amount (15 g) of polypropylene (PP) was placed in a crusher (Zamak, Mercator, Viscomix VM-30, Skawina, Poland) and then crushed and kneaded at 160 °C for 2 min. After this time, an appropriate amount (7 mL) of the concentrated solution was added dropwise to the already plasticized PP, and the material was still kneaded to mix the components for 2 min. After this time, the formed composite was placed on a press table (Fentijne Press, LabEcon 300, Utrecht, The Netherlands) and kneaded at 200 kN for 10 s at 160 °C to create a thin foil. The developed thin film was cooled down. The procedure was used for all synthesized solutions.

### 2.6. Antimicrobial Activity

The antibacterial and antifungal activities of the samples of composites made of polymer—polypropylene (PP) and nanoparticles prepared through chemical synthesis and ‘green chemistry’ against Gram-positive (*Staphylococcus aureus* strain ATCC 6538 and *Bacillus cereus* strain ATCC 10987) and Gram-negative (*Escherichia coli* strain ATCC 11229) bacteria, and fungi (*Candida krusei* strain ATCC 30135) were assessed by measuring the inhibition zones obtained from the disk diffusion assay. Microorganisms stored at −80 °C in LB broth supplemented with glycerol (a ratio of 1:1) were inoculated onto nutrient agar (bacteria) or Sabouraud agar (fungi) and incubated overnight at 37 °C. One colony of the microorganisms was then reinoculated into LB broth and cultured overnight at 37 °C with gentle shaking. The cultures were suspended to a final optical density of 0.2–0.3 at 600 nm using a V-670 spectrophotometer (Jasco International Co., Tokyo, Japan). A volume of 100 μL of the suspension was placed onto agar and spread evenly with a smoothing plunger. Composites formed as foil were aseptically cut into discs with a diameter of 8 mm and placed on the agar surface. The microorganisms were incubated at 37 °C overnight. The antibacterial and antifungal activity of the composites was assessed visually after incubation based on the inhibition of microbial growth around and under the discs. Microorganism cultures with foil discs without composites on the medium surface were considered as a control. All the experiments were carried out in quadruplicate. The microbiological media used in the study were supplied by Oxoid Ltd. (Basingstoke, UK).

## 3. Results and Discussion

### 3.1. Transmission Electron Microscopy (TEM)

**Figure 2:**
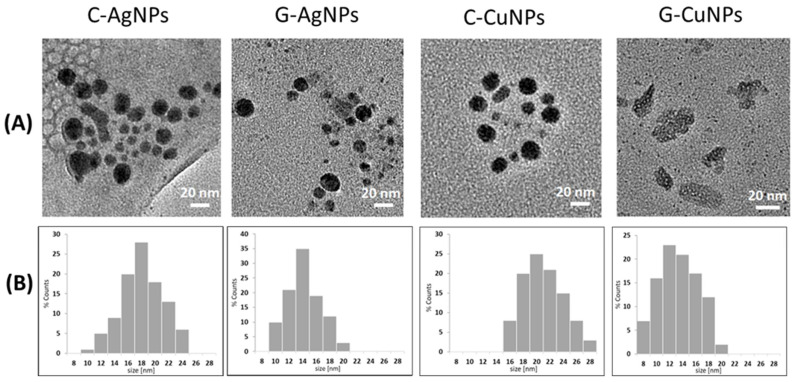
TEM images of the obtained nanoparticles (**A**); Respective histograms (**B**).

Details of the morphology and size of the synthesized nanoparticles were recorded using transmission electron microscopy and presented in Figure 2A. TEM images confirm the presence of nanoparticles of sizes and shapes dependent on the methods used during their synthesis. In frame B, a set of representative histograms is plotted. Based on the qualitative and quantitative analysis of the TEM images, the average diameters of the obtained nanostructures were calculated, their degree of agglomeration was determined, and the size distribution was assessed. For details, see Figure 2A and Table 2.

The TEM analysis revealed that the chemically synthesized silver nanoparticles (C-AgNPs) have an average size of 18 ± 3 nm. In contrast, the green-synthesized silver nanoparticles (G-AgNPs) are smaller, with an average size of 14 ± 2 nm. In the case of Ag, the homogeneity of nanoparticles is not perfect, but the particles are much more similar in size. The most homogeneous nanoparticles were obtained by chemical reduction of copper nanoparticles (see Figure 2A, C-CuNPs). ‘Green chemistry’ based synthesis of Cu results in uneven objects (see Figure 2A, G-CuNPs). For copper nanoparticles, the chemically synthesized CuNPs (C-CuNPs) showed an average size of 20 ± 3 nm, whereas the green-synthesized CuNPs (G-CuNPs) were slightly larger, averaging 13 ± 4 nm. The average diameter of the nanoparticles was estimated based on the TEM images using the commercial ImageJ IJ 1.46r program [32]. The numerical values obtained for each solution are summarized in Table 2.

Copper nanoparticles obtained in the presence of beetroot extract have particles consisting of leaf-like shapes with a rough surface and internal voids. According to the literature, similar polycrystalline images were also seen in TEM images in other Cu nanoparticles [10].

**Figure 3:**
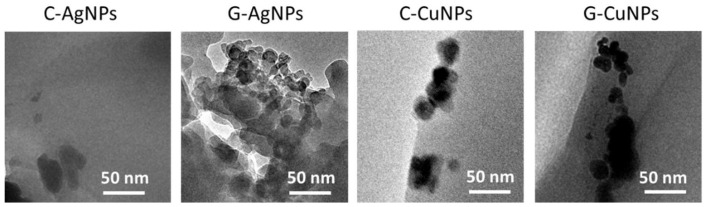
TEM images of a polymer with embedded silver and copper nanoparticles.

TEM measurements were performed to prove composite fabrication and successful incorporation of nanoparticles into the polymer (Figure 3).

The TEM images in Figure 3 show that C-AgNPs, G-AgNPs, C-CuNPs, and G-CuNPs are in the bulk of the polymer. Silver nanoparticles tend to agglomerate much more strongly (regardless of the synthesis method), whereas individual copper nanoparticles are more easily stabilized and remain better dispersed.

### 3.2. Scanning Electron Microscopy (SEM)

**Figure 4:**
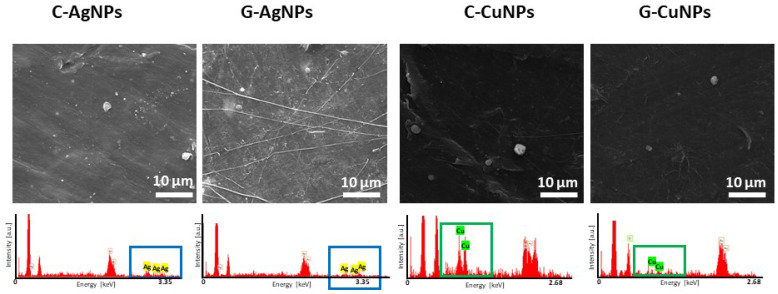
SEM images and EDX spectra of the polymer with embedded silver and copper nanoparticles, respectively.

Details of the morphology of resultant polymer-based nanoparticles-modified composites were registered using scanning electron microscopy. The obtained images are depicted in Figure 4 together with EDX spectra verifying the presence of nanoparticles.

SEM analysis reveals a relatively smooth and compact polymer matrix with randomly distributed brighter contrast regions, which can be attributed to agglomerates or clusters of embedded metallic nanoparticles. The brighter spots observed in the micrographs arise from the higher atomic number of silver and copper compared to the polymeric matrix composition, resulting in increased electron backscattering.

Presented SEM images show a random distribution of particle clusters in the matrix (brighter spots). Verification of elemental composition done by EDX confirms the composite warehouse (boxes highlight, respectively, Ag and Cu signals).

SEM analysis proves the concept of obtaining a composite of a polymer matrix with distributed particles.

### 3.3. X-Ray Diffraction (XRD)

**Figure 5:**
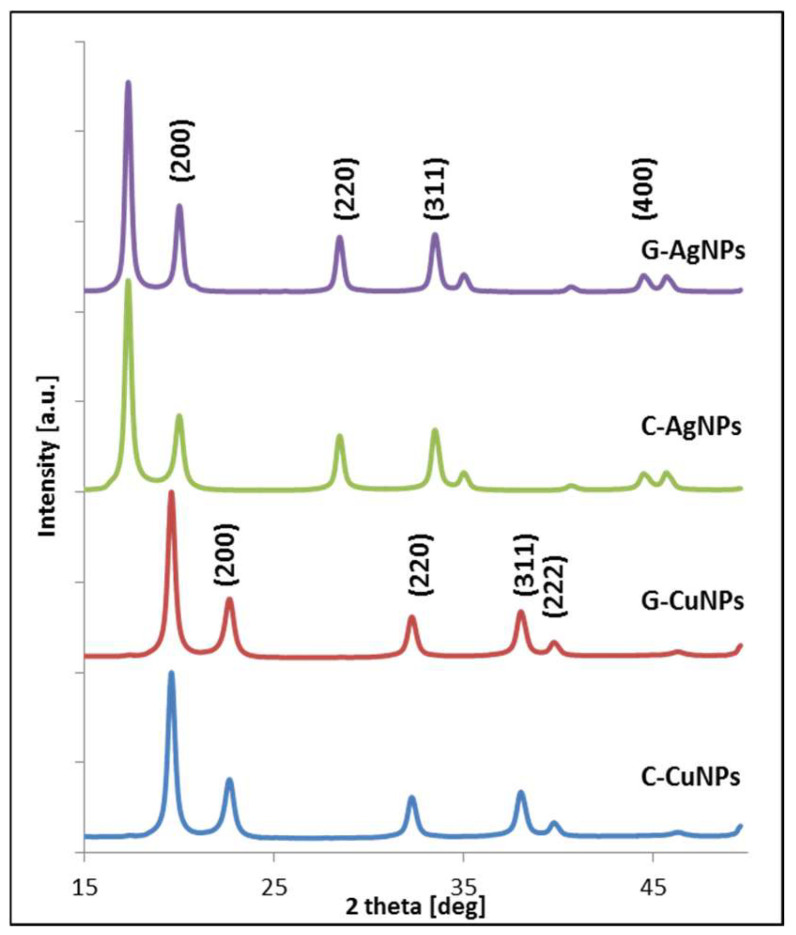
Diffractograms of synthesized Ag and Cu nanoparticles, respectively (C—chemical reduction, G—‘green chemistry’ reaction).

The crystal structure and size of the synthesized nanoparticles were identified using X-ray diffraction. The symmetry of the crystal structure of the investigated nanoparticles was determined based on diffraction patterns. The qualitative analysis of the diffraction patterns allowed us to conclude that silver and copper nanoparticles were formed in the respective reactions in forms observed in bulk [33]. Qualitative XRD patterns analysis presented in Figure 5 of the synthesized nanoparticles allows us to identify diffraction peaks from two crystallographic systems: metallic silver and metallic copper.

Quantitative evaluation of the obtained diffractograms can be done with the Williamson–Hall Equation (1), confirming the synthesis of nanoparticles of various sizes [34].

(1) $$\beta cos\theta =\left(\frac{0.9\lambda }{D}\right)+\left(4\varepsilon sin\theta \right)$$

where

*D*—grain size [Å],

*λ*—wavelength (for Mo source is 0.7136 Å),

*β*—full width at half maximum intensity of the peak [rad],

*ε*—strain,

*θ*—diffraction angle [rad].

The structure of metallic silver has fcc symmetry with the 225 space group number [35]. The experimentally determined silver lattice parameter oscillates between 4.062 and 4.072 ± 0.002 Å, in good agreement with the literature values [36]. The diffraction peaks from chemical reduction synthesis are located on the two theta axis in positions 17.01°, 19.93°, 28.35°; 33.35°, 34.53°, and 44.21°, which corresponds to the crystal hkl planes according to Miller’s nomenclature: (111), (200), (220), (311), (400) [33].

X-ray diffraction pattern analysis also revealed typical patterns for the metallic Cu fcc crystal structure, which can be indexed as (111), (200), (220), (311), (222), (400), and (331) [37]. The experimentally determined copper lattice parameter oscillates between 3.599 and 3.611 ± 0.002 Å, in good agreement with the literature values [36]. This confirms the high crystallinity and structural purity of the synthesized copper nanoparticles. The patterns’ angle positions are, respectively, equal: 19.5°, 22.43°, 32.03°, 37.78°, and 39.74°. That data is consistent with published results [11].

The crystallites’ average size of the synthesized nanoparticles and the lattice parameter and phase fraction were determined by fitting the diffraction data using the Williamson-Hall Equation (1) [34]. Where the analysis of width of the diffraction peaks at half-maximum allows the size of the nanoparticles’ crystallites to be determined. The obtained quantitative data are summarized in Table 3.

The particle sizes obtained from XRD are in good agreement with those estimated by TEM analysis, showing comparable average values for all investigated samples.

### 3.4. UV-Vis Spectroscopy

**Figure 6:**
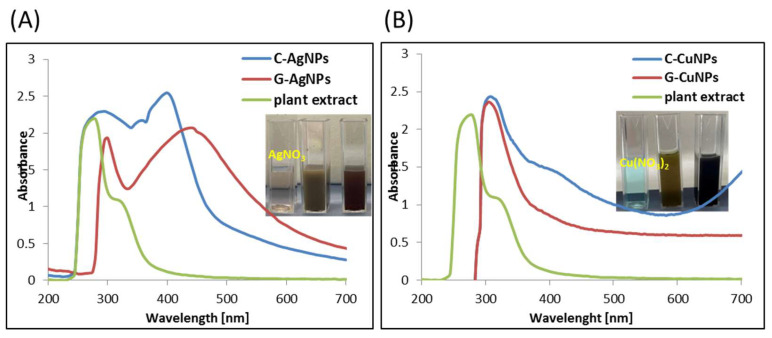
UV-Vis spectra of silver (**A**) and copper (**B**) nanoparticles obtained by chemical reduction (blue line), in the presence of plant extract (red line), and the plant extract.

UV-Vis spectroscopy measures the extinction (scattering + absorption) of light passing through a sample of Ag or Cu. Nanoparticles have unique optical properties sensitive to size, shape, concentration, state of aggregation, or refractive index near the nanoparticle surface, making UV-Vis a valuable tool for identifying and characterizing nanomaterials. In Figure 6, the UV-Vis spectra of each solution are presented in series Ag or Cu.

The characteristic absorption band associated with the surface plasmon resonance (SPR) of AgNPs is observed above 400 nm (Figure 6A), confirming the formation of silver nanoparticles [38]. According to the literature, the SPR band of metallic silver typically appears above 390 nm [39]. Therefore, absorption features observed below 390 nm cannot be attributed to the plasmon resonance of AgNPs. Importantly, similar signals are also present in chemically synthesized nanoparticles, indicating that these bands are not specific to the use of plant extract. The absorption observed below 390 nm is more likely related to the presence of organic impurities, such as residual solvents, unreacted precursors, or stabilizing agents, rather than to the nanoparticles themselves. In the case of green synthesis using beetroot extract, these signals may additionally originate from phytochemicals such as betalains, flavonoids, polyphenols, or sugars, which can act as reducing and capping agents during nanoparticle formation. These compounds may remain adsorbed on the nanoparticle surface or dispersed in the colloidal solution, contributing to absorption in the UV region. The shift in the SPR band above 400 nm toward longer wavelengths confirms nanoparticle formation. Moreover, a red shift is commonly associated with an increase in particle size [40].

The UV-Vis absorption spectra shown in Figure 6B display characteristic SPR features of CuNPs. A maximum absorbance observed around 320 nm indicates the presence of copper nanoparticles in both samples [41], which is consistent with previously reported data for CuNPs [42]. Minor shifts in the absorption maximum can be attributed to differences in particle size. The broad band observed around 400 nm is likely associated with organic compounds and/or solvent residues present in the samples rather than with the SPR of CuNPs.

### 3.5. Dynamic Light Scattering (DLS)

**Figure 7:**
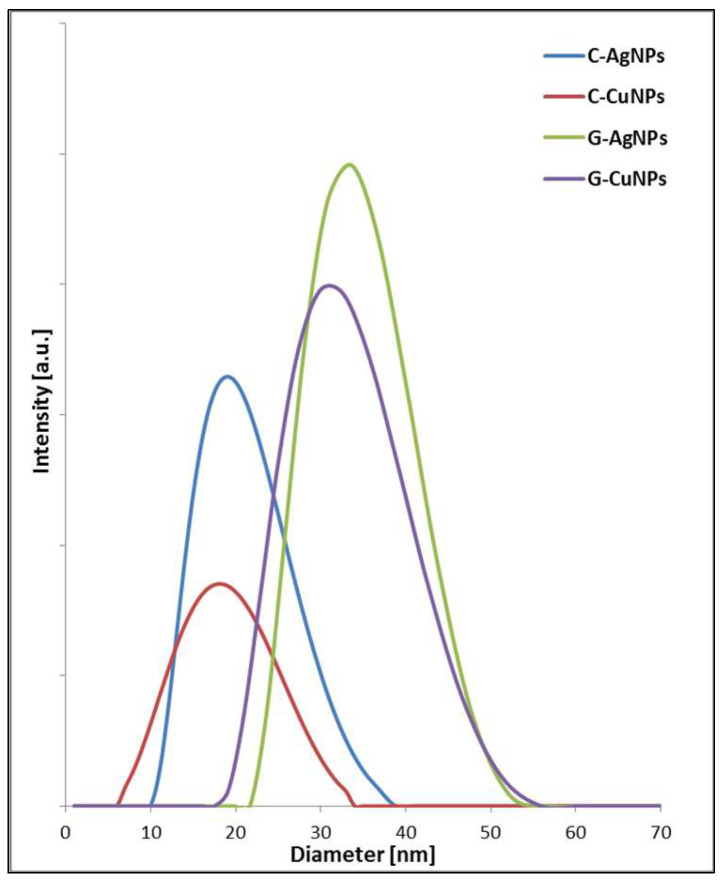
DLS data of the nanoparticles fabricated with chemical reagents (chemical reduction) and beetroot extract.

The DLS measurements were conducted to determine the average hydrodynamic size obtained in a given solution of nanoparticles, their size distribution, and their tendency to aggregate. In the solutions of both silver and copper nanoparticles obtained in the presence of beetroot extract, narrow peaks were recorded, indicating a small size distribution of the obtained nanostructures (see Figure 7). To correctly interpret the results of DLS analyses, choosing the appropriate way of presenting them is necessary. Inadequate processing may cause the presence of a few volume percent of larger nanoparticles to overshadow the number of smaller ones completely [43].

Qualitative analysis of the DLS spectra allows us to conclude that the nanoparticles obtained due to chemical reduction are consistent with those obtained in TEM imaging. The size of particles registered for green synthesis is in both cases larger than what is related to the presence of an organic corona on the particle’s surface. The size distribution of the identified objects in individual samples is associated with the variable hydrodynamic radius, which is related to other organic layers formed around the inorganic core [44].

The estimated size by simulation is defined for a sphere model with the same diffusion coefficient as the measured nanostructure. As a result, all this may cause nanoparticles’ diameter to differ significantly from that determined using other methods, such as TEM. Therefore, the DLS measurement identifies more than the core of nanoobjects. It identifies the structure as a whole. In addition, suppose the particles tend to agglomerate, i.e., the system is dynamic, it is recorded in subsequent measurements that there are more and more particles with increasing hydrodynamic radius [45]. Both series of samples, Ag and Cu, show the same tendency (Figure 7). The discrepancy between particle sizes measured by TEM and DLS for green-synthesized AgNPs and CuNPs is due to the presence of organic molecules from the beetroot extract capping the nanoparticles [46]. TEM measures the core size, while DLS measures the hydrodynamic diameter, including the organic layer and solvent shell [45]. Chemically synthesized nanoparticles lack this organic coating, resulting in better agreement between TEM and DLS sizes [44,46].

Smaller nanoparticles exhibit higher antibacterial activity due to larger surface area, enhanced ion release, and stronger membrane interaction. DLS data additionally show dispersion behaviour in aqueous conditions relevant to antibacterial assays. XRD confirms crystalline phase and purity. Since different crystallographic phases of Ag, and Cu, nanoparticles release metal ions at different rates, XRD provides essential structural information explaining variations in antibacterial performance.

### 3.6. Antimicrobial Effect of Polymers Containing Nanoparticles

**Figure 8:**
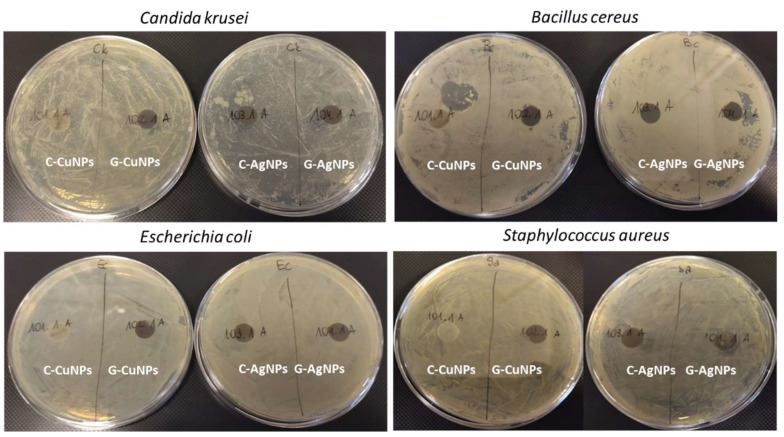
Disc diffusion for a polymer with embedded nanoparticles.

Disk diffusion tests were performed to check the antimicrobial properties of the obtained nanocomposites containing polymer and nanoparticles (Figure 8). Each composite type was subjected to the pathogens, and after 24 h, the inhibitory effect was checked visually. The microorganisms were selected as standard Gram-positive, Gram-negative and fungal reference strains widely used in antimicrobial nanomaterial testing.

As an additional control, we also tested the polypropylene matrix without nanoparticles. The pure PP material did not exhibit any antibacterial or antifungal activity, confirming that the polymer itself has no intrinsic antimicrobial properties. In the case of a composite with both copper and silver nanoparticles obtained with the ‘green chemistry’ (right side of the plates), a zone of inhibition of the microorganism tested growth under the discs was noted. Similarly, the composites with silver nanoparticles obtained through the chemical synthesis inhibited the microbial growth under the discs. However, the composites with copper nanoparticles synthesized similarly (chemical synthesis) showed no inhibitory effect on the microorganisms tested.

The obtained polymers with copper nanoparticles can be applied to materials such as agro textiles. Due to its valuable properties, polyvinylpyrrolidone (PVP) is often used in biomedical applications [47]. For instance, adding this polymer to drug carriers significantly increases the efficiency of their assimilation by organisms and extends their resistance time in the bloodstream [48]. Moreover, magnetic nanoparticles coated with poly(ethylene glycol) are also used in anticancer heat therapy, during which cancerous cells are destroyed in a controlled manner [49]. Polymer nanocomposites containing metal nanoparticles are a group of materials with high application potential and extend the scope of use of plastics as materials [50].

Copper ions interact with bacterial cells by binding to negatively charged cell wall and membrane components, disrupting membrane integrity. Furthermore, Cu^2+^ can penetrate bacterial cells and catalyze the formation of reactive oxygen species (ROS) through Fenton-like reactions, leading to oxidative damage of DNA, proteins, and lipids. The generation of oxidative stress ultimately compromises vital cellular processes, leading to bacterial cell death [49]. The second mechanism involves direct contact between Cu NPs and bacterial surfaces, where nanoparticles may adhere to the membrane, induce physical damage or destabilization, and increase permeability [50]. In some cases, internalization of nanoparticles may occur, further disrupting intracellular function.

Research shows that green-chemistry-prepared copper nanoparticles have better results than chemically reduced ones when testing their inhibitory activity. Gram-positive bacteria have one membrane with a multi-layered peptidoglycan polymer and a thick cell wall. In contrast, Gram-negative bacteria have two membranes with a fragile polymer. In our study, copper nanoparticles exhibited antibacterial activity against Gram-negative *E. coli* (ATCC 25922), producing a clear inhibition zone, whereas the copper salt solution (Cu^2+^ ions), used as a non-nanoparticle control, showed significantly lower activity.

According to the literature, small-sized particles penetrate the bacterial cell well and have a better inhibitory effect than on Gram-positive bacteria [51]. The distinct activity is due to differences in particle size and phytochemicals in the plant extract. When the synthesized nanoparticles have a negative charge that strongly binds to the negatively charged microorganism, the concentration of oxygen increases as a result of this excitation [52]. Singlet oxygen is more reactive and enhances the antibacterial effect [52]. In the presented case, we must also consider the specific particle behavior related to the composite form. Copper nanoparticles are widely incorporated into polymeric films due to their excellent antimicrobial and functional properties. However, copper nanoparticles may exhibit cytotoxic or ecotoxic effects at elevated concentrations, primarily due to the release of copper ions (Cu^2+^). Therefore, evaluating both the extent and the rate of nanoparticle or ion leaching from the films is crucial under realistic conditions. Such assessment is essential to ensure the safety of the final material, particularly in applications involving direct contact with food, biological tissues, or the environment. According to the European Food Safety Authority (EFSA), the tolerable upper intake level (UL) for copper is 5 mg/day for adults. At the same time, the U.S. Environmental Protection Agency (EPA) has set the maximum contaminant level goal (MCLG) for copper in drinking water at 1.3 mg/L. These values provide reference thresholds against which the leaching behavior of Cu-containing films should be evaluated. Ensuring that the release of copper remains well below these limits is critical to minimize potential toxicological risks and to comply with regulatory requirements.

To support the mechanistic interpretation presented in the antimicrobial activity section, it is important to emphasize that the behaviour observed in our study is consistent with well-established mechanisms reported in the literature [53,54]. Numerous studies have demonstrated that green-synthesized metal nanoparticles exhibit enhanced antimicrobial effects due to the combined influence of their small crystallite size, reduced agglomeration, and the presence of phytochemical residues acting as stabilizers and electron donors [53,54,55]. These phytochemicals (e.g., phenolics, flavonoids, betalains present in beetroot extract) are known to facilitate ROS generation and increase the rate of metal-ion release, which directly correlates with enhanced antimicrobial potency [56]. Furthermore, previous reports have shown that copper nanoparticles produced via green chemistry generate higher levels of reactive oxygen species than chemically reduced particles of similar nominal size, confirming the size and surface chemistry-dependent nature of their activity [57]. In addition, studies on polymer-nanoparticle composites indicate that antimicrobial effects depend strongly on nanoparticle accessibility and surface exposure [58]. The inhibition zones observed for PP composites containing green Ag and Cu nanoparticles align with principle: the nanoparticles produced via the plant-extract route retain higher surface reactivity and interaction potential even after incorporation into the polypropylene matrix [59]. In contrast, chemically synthesized copper nanoparticles, known to exhibit lower redox activity and stronger agglomeration, show diminished or absent antimicrobial effects when embedded in polymers, which is consistent with our findings [60]. Green-synthesized nanoparticles showed stronger effects due to their smaller size, reduced agglomeration, and phytochemical capping layers, which enhanced Cu^2+^/Ag^+^ release and ROS generation. Gram-negative *E. coli* was particularly sensitive to green Cu NPs, while chemically synthesized Cu NPs showed no inhibition. PP without nanoparticles produced no activity, confirming that antimicrobial effects originate solely from the incorporated nanoparticles. Antibacterial activity can be quantified using MIC/MBC or inhibition-zone measurements.

In this study, green-synthesized Ag and Cu nanoparticles showed smaller and more uniform particle sizes, higher stability (TEM/DLS/UV-Vis), and stronger bactericidal/fungicidal activity compared to chemically synthesized nanoparticles, confirming their superior antimicrobial performance.

## 4. Conclusions

This study demonstrates that copper and silver nanoparticles synthesized via a green chemistry approach using beetroot extract can be successfully incorporated into a polypropylene polymer matrix while preserving their physicochemical integrity and antimicrobial functionality. Notably, the composites containing green-synthesized Cu nanoparticles exhibited significantly enhanced bactericidal and fungicidal properties compared to silver-based composites and copper nanoparticles obtained by conventional chemical reduction. These results confirm that the synthesis routine plays a decisive role in dictating the biological activity of the resulting polymer nanocomposites. The superior antimicrobial efficiency of green-synthesized Cu nanoparticles highlights their potential as effective, sustainable alternatives to conventionally produced nanomaterials in applications where resistance to bacterial or fungal growth is critical. By maintaining structural stability within the polymer matrix, these nanoparticles retain their active functionality and open promising avenues for developing antimicrobial packaging, agricultural films, and biomedical materials. Taken together, our findings validate the feasibility of introducing green-synthesized nanoparticles into polymer systems and provide strong evidence that the choice of synthesis method directly influences the biological performance of the resulting nanocomposites. Future work should focus on scaling the synthesis approach, assessing long-term stability, and exploring application-specific optimization, thereby bridging laboratory advances with industrial and clinical applications.

## Figures and Tables

**Table 1:** Representation of sample names.

Sample	Synthesis
**C-AgNPs**	chemical reduction (NaBH_4_ + SDS)
**G-AgNPs**	‘green chemistry’ (beetroot extract)
**C-CuNPs**	chemical reduction (NaBH_4_ + SDS)
**G-CuNPs**	‘green chemistry’ (beetroot extract)

**Table 2:** Numerical values of the obtained nanoparticles.

Sample	Size (TEM) [nm]	UV-Vis Absorbance Maximum ± 5 [nm]	DLS Size [nm]
**C-AgNPs**	18 ± 3	405	18 ± 4
**G-AgNPs**	14 ± 2	425	32 ± 5
**C-CuNPs**	20 ± 3	320	15 ± 3
**G-CuNPs**	13 ± 3	321	30 ± 4

**Table 3:** The size of crystallites and lattice parameters were calculated from XRD data.

Sample	Size (XRD) [nm] ±2	Lattice Parameter (Ag) Å ± 0.002	Lattice Parameter (Cu) Å ± 0.02
**C-AgNPs**	19	4.072	-
**G-AgNPs**	13	4.069	-
**C-CuNPs**	18	-	3.611
**G-CuNPs**	15	-	3.599

## Data Availability

The original contributions presented in this study are included in the article. Further inquiries can be directed to the corresponding author.
